# Quality of life of Venezuelan migrants in Brazil during the COVID-19 pandemic

**DOI:** 10.1590/0102-311XEN147423

**Published:** 2024-06-21

**Authors:** Iaralyz Fernandes Farias, Anete Trajman, Paulo Nadanovsky, Manuel Ribeiro, Eduardo Faerstein

**Affiliations:** 1 Instituto de Medicina Social Hesio Cordeiro, Universidade do Estado do Rio de Janeiro, Rio de Janeiro, Brasil.; 2 Universidade Federal do Rio de Janeiro, Rio de Janeiro, Brasil.; 3 McGill University, Montreal, Canada.; 4 Escola Nacional de Saúde Pública Sergio Arouca, Fundação Oswaldo Cruz, Rio de Janeiro, Brasil.

**Keywords:** Human Migration, COVID-19 Pandemic, Quality of Life, Global Health, Migración Human, Pandemia de COVID-19, Calidad de Vida, Salud Global, Migração Humana, Pandemia por COVID-19, Qualidade de Vida, Saúde Global

## Abstract

The economic, social, and health crisis in Venezuela has resulted in the largest forced migration in recent Latin American history. The general scenario in host countries influence migrants’ self-perception of quality of life, which can be understood as an indicator of their level of integration. The COVID-19 pandemic has exacerbated socioeconomic and health vulnerabilities, especially for forced migrants. We hypothesized that the adverse circumstances faced by Venezuelan migrants during the pandemic have deepened their vulnerability, which may have influenced their perception of quality of life. This study aims to evaluate the quality of life of Venezuelan migrants in Brazil during the COVID-19 pandemic. We assessed the quality of life of 312 adult Venezuelan migrants living in Brazil using the World Health Organization WHOQOL-BREF quality of life assessment, which was self-administered online from October 20, 2020, to May 10, 2021. The associations of quality of life and its domains with participants’ characteristics were analyzed via multiple linear regression models. Mean quality of life score was 44.7 (±21.8) on a scale of 0 to 100. The best recorded mean was in the physical domain (66.2±17.8) and the worst in the environmental domain (51.1±14.6). The worst quality of life was associated with being a woman, not living with a partner, lower household income, and discrimination based on nationality. Factors associated with overall quality of life and respective domains, especially income and discrimination, were also observed in other studies as obstacles to Venezuelan migrants. The unsatisfactory quality of life among Venezuelans living in Brazil may have been worsened by the pandemic during the study period.

## Resumen

La crisis económica, social y sanitaria en Venezuela ha provocado la mayor emigración forzada en la historia reciente de América Latina. Las circunstancias imperantes en los países de acogida influyen en la calidad de vida autopercibida de los inmigrantes, lo que puede entenderse como un indicador de su nivel de integración. La pandemia del COVID-19 ha intensificado las vulnerabilidades socioeconómicas y sanitarias, especialmente para los inmigrantes forzados. Se plantea la hipótesis de que las circunstancias adversas que enfrentaron los inmigrantes venezolanos durante la pandemia profundizaron su vulnerabilidad, lo que puede haber influido en su percepción de la calidad de vida. Este estudio tiene como objetivo evaluar la calidad de vida de los inmigrantes venezolanos en Brasil durante la pandemia del COVID-19. Se evaluó la calidad de vida de 312 inmigrantes venezolanos adultos viviendo en Brasil mediante una herramienta desarrollada por la Organización Mundial de la Salud (WHOQOL-BREF), autollenada en línea del 20 de octubre de 2020 al 10 de mayo de 2021. Las asociaciones de la calidad de vida y sus dominios con las características de los participantes se analizaron mediante modelos de regresión lineal múltiple. La puntuación media de la calidad de vida fue de 44,7 (±21,8) en una escala de 0 a 100. La mejor media registrada fue en el dominio físico (66,2±17,8), y la peor en el dominio medio ambiente (51,1±14,6). La peor calidad de vida se asoció al sexo femenino, no convivir con pareja, menores ingresos y discriminación por nacionalidad. Los factores asociados con la calidad de vida general y sus respectivos dominios, especialmente ingresos y discriminación, coinciden con lo presentado por otros estudios como obstáculos para los inmigrantes venezolanos. La calidad de vida insatisfactoria entre los venezolanos que viven en Brasil debió de ser empeorada por la pandemia en el período estudiado.

## Resumo

A crise econômica, social e sanitária na Venezuela resultou na maior emigração forçada da história recente da América Latina. As circunstâncias prevalentes nos países de acolhimento influenciam a autopercepção da qualidade de vida dos imigrantes, o que pode ser entendido como um indicador do seu nível de integração. A pandemia da COVID-19 exacerbou as vulnerabilidades socioeconômicas e de saúde, especialmente para imigrantes forçados. Levantamos a hipótese de que as circunstâncias adversas enfrentadas pelos imigrantes venezuelanos durante a pandemia aprofundaram sua vulnerabilidade, o que pode ter influenciado sua percepção de qualidade de vida. Este estudo tem como objetivo avaliar a qualidade de vida de imigrantes venezuelanos no Brasil durante a pandemia da COVID-19. Avaliamos a qualidade de vida de 312 imigrantes venezuelanos adultos vivendo no Brasil por meio de um instrumento desenvolvido pela Organização Mundial da Saúde (WHOQOL-BREF), autoadministrado *online* de 20 de outubro de 2020 a 10 de maio de 2021. As associações da qualidade de vida e seus domínios com as características dos participantes foram analisadas por meio de modelos de regressão linear múltipla. O escore médio de qualidade de vida foi de 44,7 (±21,8) em uma escala de 0 a 100. A melhor média registrada foi no domínio físico (66,2±17,8) e a pior no domínio do meio ambiente (51,1±14,6). A pior qualidade de vida associou-se ao sexo feminino, não viver com companheiro, menor renda familiar e discriminação por nacionalidade. Fatores associados à qualidade de vida geral e seus respectivos domínios, especialmente renda e discriminação, também foram observados em outros estudos como obstáculos aos imigrantes venezuelanos. A qualidade de vida insatisfatória entre os venezuelanos residentes no Brasil pode ter sido agravada pela pandemia no período estudado.

## Introduction

In the largest forced migration in recent Latin American history, 6.9 million Venezuelans emigrated from 2017 to October 2023 due to the political and socioeconomic crisis in their home country, and Brazil was one of the main host countries, especially in the Global South ^1^. By November 2023, 510,100 Venezuelans were living in Brazil ^2^.

Forced migration is any situation in which individuals are forced to leave their usual place of residence due to reasons beyond their desire or interest ^3^. Although the United Nations High Commissioner for Refugees (UNHCR) considers that the reasons for the Venezuelan exodus do not fit the definition of refugees in the Refugee Statute ^4^. That agency argues that the circumstances that led to the outflow would fit the broader definition of a refugee in the Cartagena Declaration, with an irrefutable need for international protection ^5^ ^,^ ^6^. In Brazil, Venezuelan visas were validated temporarily for humanitarian reception ^7^ ^,^ ^8^.

Quality of life can be understood as an important indicator of migrants’ integration in the host country, with several physical, psychological, social, and environmental predictors that can negatively affect their perception, such as feeling discriminated, difficulty in accessing health services, unemployment, and weak social network ^9^ ^,^ ^10^. The quality of life of forced migrants is generally lower than that of the overall population in host countries ^11^. They tend to be more satisfied with their lives in host countries that have lower levels of economic inequality, good public services, and a safer, more welcoming social environment ^12^. The support of organizations and institutions for access to services relevant to their reception and stay also contributes to the integration process ^13^. Furthermore, the time spent living in the host country tends to improve the socioeconomic condition and quality of life of migrants due to their gradual integration ^10^ ^,^ ^14^.

The World Health Organization (WHO) defines quality of life as a broad, multidimensional concept that includes an individual’s subjective assessment of their physical health, psychological state, level of independence, social relationships, personal beliefs, and their relationship with the environment ^15^. Thus, quality of life reflects the perception of individuals that their needs are being satisfied or that they are being denied opportunities to achieve happiness and self-realization, regardless of their physical state of health or social and economic conditions ^16^. Although the concept of quality of life is complex and subjective, different tools were created to quantify it ^17^, e.g., the WHOQOL instruments, to allow comparison and progression of some specific aspects of quality of life.

The COVID-19 pandemic has amplified the inequalities and vulnerabilities of migrants worldwide, favoring processes of social exclusion, also noted in the South America and the Southern Cone ^18^ ^,^ ^19^. In Brazil, where migrants may transit or stay, the pandemic created severe work, income, education, and health difficulties ^20^, resulting from an increase in unemployment and informal jobs, income shortage, and lack of knowledge regarding access to education and health institutions ^21^.

To the best of our knowledge, no studies have been conducted on the quality of life of Venezuelan migrants in Brazil. We hypothesized that the adverse circumstances faced by Venezuelan migrants during the COVID-19 pandemic have deepened their vulnerability, which may have negatively influenced their perception of quality of life. This study aims to evaluate the quality of life of Venezuelan migrants in Brazil during the COVID-19 pandemic.

## Methods

This study was approved by the Research Ethics Committee of the State University of Rio de Janeiro (UERJ; CAAE: 31909220.1.0000.5260); WHO authorized the use of the WHOQOL-BREF (identification: 361703); and participants signed an online consent form.

This is a cross-sectional study that used an online self-administered questionnaire via the REDCap platform (https://redcapbrasil.com.br/). Venezuelans aged 18 years or older living in Brazil and who answered at least 80% of the quality of life questionnaire were included. Recruitment strategies included: invitation participants of an online Portuguese course administered by UERJ and Cáritas-RJ; referral from the Sérgio Vieira de Mello Academic Chairs of Brazilian universities; and snowball sampling on Facebook, Instagram, Telegram, and WhatsApp. Invitations reached 577,247 individuals. Among 327 respondents, 18 years or older, 15 were excluded, based on: failure to sign the consent form, limited understanding of the questionnaire, illiteracy, or answering fewer than 80% of the items in the questionnaire.

After a pilot study conducted in 2020, the questionnaire in Spanish was applied from October 20, 2020, to May 10, 2021, addressing three modules: sociodemographic characteristics, migration aspects, and quality of life. The first module included 17 questions and the second 13 questions, both of which could be redirected to complementary questions. To evaluate the qualty of life, the Spanish version of the WHOQOL-BREF ^22^ was adopted, with 26 questions: two extra-domain items (health and overall quality of life) and 24 facets subdivided into four domains (physical, psychological, social relations, and environment).

The WHOQOL-BREF instrument is a shorter version of the WHOQOL-100, both created by the WHO in a multicenter field work ^15^. Although Brazil was one of the centers where the instrument was created and tested, studies conducted with people from or living in Spanish-speaking countries, without their own version of the WHOQOL-BREF, opted to use the Spanish version ^23^ ^,^ ^24^. Each item of the WHOQOL-BREF is rated by a Likert scale ranging from 1 (very dissatisfied) to 5 (very satisfied). Following recommendations by the WHO, this study analyzed the answers to the WHOQOL-BREF with scores converted to a scale from 0 to 100 (worst and best possible scores, respectively).

The frequencies and 95% confidence intervals (95%CI) of variables related to sociodemographic and migration profile were estimated, as well as mean scores and standard deviations of overall quality of life, facets, domains, and Cronbach’s α, standardized for each domain. Missing data were not included in the calculation of relative frequencies. Multiple linear regression models were estimated for the analyses of the associations of overall quality of life and its domains (dependent variables) with the profile (independent variables) of the respondents. The independent variables initially selected were those reported in the literature and that presented p-values < 0.20 in the bivariate analyses of the quality of life scores. Independent variables with p-values < 0.05 remained in the models, using the backward stepwise strategy. The studentized residuals of the models confirmed most linear regression assumptions such as normality, absence of outliers, multicollinearity, and homoscedasticity. The R software version 3.4.2 (http://www.r-project.org) was used.

## Results

Among the 312 Venezuelan migrants included, 65.7% were women, median age was 36 years (interquartile range - IQR: 29-44), 54.2% had 15 to 19 years of schooling, and 52.4% were married or living in a stable union. In total, 58% of the participants had no paid activity, 14.8% reported no household income, and 27.2% received up to USD 92 of monthly household income. Moreover, 34% of the Venezuelans participating in the study had experienced nationality-based discrimination, 38.1% had a fixed-term residence permit, and 37.5% lived in Northern Brazil (Table 1).

**Table 1:** Sociodemographic and migratory profile of Venezuelans living in Brazil, 2020-2021.

Sociodemographic and migratory characteristics	n	%	95%CI
Sex			
Female	205	65.7	60.1; 70.9
Male	107	34.3	29.1; 39.9
Total	312	100.0	-
Age group (years)			
18-29	82	26.3	21.6; 31.6
30-39	109	34.9	29.7; 40.5
40-49	73	23.4	18.9; 28.6
50 or more	48	15.4	11.7; 20.0
Total	312	100.0	-
Schooling (years of schooling)			
Never attended school or < 1	10	3.2	1.6; 6.0
1-7	7	2.3	1.0; 4.8
8-10	3	1.0	0.3; 3.0
11-14	83	26.8	22.0; 32.1
15 or more	207	66.8	61.2; 71.9
Missing data	2	-	-
Total	312	100.0	-
Marital status			
Married or in a stable union	162	52.4	46.7; 58.1
Single	120	38.8	33.4; 44.5
Separated/Divorced or widowed	27	8.7	5.9; 12.6
Missing data	3	-	-
Total	312	100.0	-
Monthly household income (USD) *			
No income	43	14.8	11.0; 19.6
≤ 92	79	27.2	22.3; 32.8
93-85	71	24.5	19.7; 29.9
186-277	46	15.9	12.0; 20.7
≥ 278	51	17.6	13.5; 22.6
Missing data	22	-	-
Total	312	100.0	-
Paid work in Brazil			
No	163	55.3	49.4; 61.0
Yes, part-time job	55	18.6	14.5; 23.7
Yes, up to 30 hours/week	19	6.4	4.0; 10.0
Yes, more than 30 hours/week	58	19.7	15.4; 24.8
Missing data	17	-	-
Total	312	100.0	-
Discriminated due to nationality			
Yes	105	33.7	28.5; 39.2
No	207	66.3	60.8; 71.5
Total	312	100.0	-
Migratory status			
Fixed-term residence permit	119	38.1	32.8; 43.8
Residence permit for an indefinite period	50	16.0	12.2; 20.7
Refugee	48	15.4	11.7; 20.0
Resident permit applicant	36	11.5	8.3; 15.7
Undocumented	34	10.9	7.8; 15.0
Asylum seeker	25	8.0	5.3; 11.7
Total	312	100.0	-
Region of residence in Brazil			
North	117	37.5	32.2; 43.2
Southeast	85	27.2	22.5; 32.6
South	75	24.0	19.5; 29.2
Northeast	23	7.4	4.8; 11.0
Central-West	12	3.8	2.1; 6.8
Total	312	100.0	-

95%CI: 95% confidence interval.

Source: prepared by the authors.

* The average exchange rate during the study period was USD 1 = BRL 5.42.

**Figure 1:**
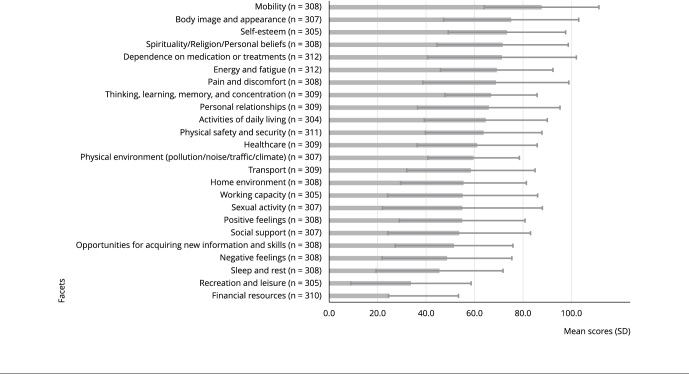
WHOQOL-BREF facets mean scores (scale 0-100) and standard deviations of Venezuelan migrants in Brazil, 2020-2021.

Overall quality of life showed a mean score of 44.7 (standard deviation - SD = 21.8) on the 0-100 scale. The facets with the highest mean scores were “mobility” (87.7) and “body image and appearance” (75.1). The lowest were “financial resources” (24.8) and “recreation/leisure” (33.8) (Figure 1). The physical domain presented the best mean assessment, with 66.2 points (SD = 17.8; α = 0.78), followed by the psychological (65.0 points; SD = 17.4; α = 0.78), social relations (58.1 points; SD = 24.2; α = 0.69), and environmental (51.1 points; SD = 14.6; α = 0.77) domains. The overall quality of life and domains variables were considered to have a normal distribution.

SD: standard deviation. Source: prepared by the authors. Note: variations in totals are due to missing data.

Low family income and having experienced discrimination reduced the overall quality of life score (reduction of up to 23.7 and 6.7 points, respectively). Among the quality of life domains, being a woman decreased the physical and psychological domains by 6.2 points, and the latter also scored 7.3 points lower among those who had experienced some episode of discrimination. In the social relations domain, lower score was also associated with discrimination, female sex, and not living with a partner. Finally, experience of discrimination resulted in a 4-point decrease in the score in the environment domain (Table 2). Variations in totals are due to missing data.

**Table 2:** Associations of general quality of life and domains with sociodemographic and migratory profile of Venezuelan migrants in Brazil, 2020-2021.

Sociodemographic and migratory characteristics	General quality of life (n = 289)		Domain							
			Physical (n = 312)		Psychological (n = 312)		Social relations (n = 309)		Environment (n = 312)	
	R^2^/R^2^ adjusted: 0.150/0.135		R^2^/R^2^ adjusted: 0.028/0.025		R^2^/R^2^ adjusted: 0.070/0.064		R^2^/R^2^ adjusted: 0.114/0.102		R^2^/R^2^ adjusted: 0.016/0.013	
	β	95%CI (p-value)	β	95%CI (p-value)	β	95%CI (p-value)	β	95%CI (p-value)	β	95%CI (p-value)
Intercept	60.9	55.3; 66.6 (< 0.001)	70.1	66.8; 73.5 (< 0.001)	71.7	68.3; 75.2 (< 0.001)	71.1	65.9; 76.4 (< 0.001)	57.9	54.3; 61.4 (< 0.001)
Monthly household income (USD) *										
≥ 278	1.0	-	-	-	-	-	-	-	-	-
No income	-23.7	-31.8; -15.7 (< 0.001)	-	-	-	-	-	-	-	-
≤ 92	-18.8	-25.8; -11.7 (< 0.001)	-	-	-	-	-	-	-	-
93-185	-15.3	-22.4; -8.1 (< 0.001)	-	-	-	-	-	-	-	-
186-277	-9.4	-17.3; -1.4 (0.021)	-	-	-	-	-	-	-	-
Discriminated due to nationality										
No	1.0	-	-	-	1.0	-	1.0	-	1.0	-
Yes	-6.7	-11.6; -1.9 (0.007)	-	-	-7.3	-11.2; -3.3 (< 0.001)	-9.0	-14.4; -3.5 (0.001)	-4.0	-7.4; -0.5 (0.024)
Sex										
Male	-	-	1.0	-	1.0	-	1.0	-	-	-
Female	-	-	-6.2	-10.4; -2.1 (0.003)	-6.2	-10.4; -2.1 (0.003)	-6.5	-11.9; -1.0 (0.020)	-	-
Marital status										
Married or in a stable union	-	-	-	-	-	-	1.0	-	-	-
Single	-	-	-	-	-	-	-11.4	-16.8; -6.0 (< 0.001)	-	-
Separated/Divorced or widowed	-	-	-	-	-	-	-13.0	-22.4; -3.6 (0.007)	-	-

95%CI: 95% confidence interval.

Source: prepared by the authors.

Note: variations in totals are due to missing data.

* The average exchange rate during the study period was USD 1 = BRL 5.42.

## Discussion

The overall quality of life of Venezuelan migrants in Brazil included in our study during the COVID-19 pandemic was perceived as lower than half of the maximum score on the multidimensional WHOQOL-BREF instrument. Physical domain scored the best; while the environment worst. Poorer quality of life (overall and in specific domains) was associated with female sex, living without a partner, low household income, and having experienced discrimination due to nationality.

The methodological approach adopted snowball sampling, as well as the use of social networks to recruit study participants. We highlight that other studies with migrant populations during the COVID-19 pandemic have also used this approach ^25^. Limitations for participation included limited access to the internet and possibly a lack of a relationship of trust ^26^ ^,^ ^27^.

The higher proportion of women in the study was consistent with the higher female presence in Venezuelan migration to Brazil. Monthly income of less than USD 93, or less than half of the Brazilian minimum wage (value in 2022: USD 224) was reported by 42% of participants; about 15% mentioned no income. The high proportion of migrants with higher education contrasts with the high frequency of very low income. In another sample of Venezuelans who entered Brazil via Roraima - a state that has common border with Venezuela - there were also reports of about half the income of Brazilians ^20^. Their apparently more severe economic vulnerability may be partly explained by the higher rate of unpaid activity. The negative impact of low income on quality of life was expected and has been reported in other migratory groups in Latin America ^19^ ^,^ ^24^.

More than one third of the respondents mentioned discrimination due to their nationality, not a surprising nor recent fact in Brazil. The country’s migration policy privileged white European migrants, with a subsequent attempt to assimilate them and their descendants to consolidate a national unity in cultural, religious, and ethnic patterns ^28^ ^,^ ^29^. This intention of “whitening” the Brazilian population is well-known and manifests itself in various ways ^30^. The several reported episodes of xenophobia also contributed to the breakdown of “Brazilian cordiality” mith ^31^. Discrimination has already been mentioned as an obstacle to integration in Brazil: among nearly 500 refugees from other countries, 41% had suffered some kind of discrimination based on their foreign nationality, race/skin color, or sexual orientation ^32^. Notably, quality of life is worse in those who experienced discrimination, having also been reported in other forced migrant groups ^33^.

Data collection occurred at the beginning of the COVID-19 pandemic, in which high mortality, severe insecurity, and an intense economic crisis occurred in Brazil, which may have worsened the quality of life of Venezuelan participants in this study, especially during the period of social isolation, due either to decreased or lost income, difficulties in securing healthcare, and postponement of decisions on their migration status ^19^ ^,^ ^34^. Thus, during the advance of COVID-19, comprehensive healthcare for migrants was further limited due to the lack of information about their rights and language difficulties ^35^. Regarding exposure to coronavirus, along with no equitable access to COVID-19 vaccines, migrants reported difficulty in adhering to social distancing measures since they had to leave home in search of financial resources. Moreover, the sometimes restricted housing structure hindered in-home isolation when necessary ^21^.

Over the last few years, Brazil has experienced a change in the origin of migrants arriving in the country - no longer predominantly from the Global North ^36^. In the context of South-South migration, the predominantly regional flow of Venezuelans highlights this reconfiguration and indicates the need to prepare for the increase in migrants from the Global South ^37^. However, at the beginning of the pandemic, migratory flows to Brazil were negatively impacted by laws that restricted access to the country with closed borders, thus undermining the right to reception provided for in national laws and treaties to which Brazil is a signatory ^38^.

The circumstances in the host country influence migrants’ self-perception of quality of life. The COVID-19 pandemic has exacerbated socioeconomic and health problems, which may have led to a worsening of quality of life among the Venezuelans who participated in the study. Factors associated with overall quality of life and its domains, especially income and discrimination, were also observed in other studies as obstacles to Venezuelans and their effective integration ^21^ ^,^ ^39^. Therefore, considering not only the implementation of intersectoral public policies, but also the Global Health scenario, the assessment of quality of life can aid monitoring progress toward achieving the Sustainable Development Goals outlined in the United Nations 2030 Agenda ^40^.

This study presents some limitations, including: uneven representation of Brazil’s states; potential recall and/or censoring biases affecting reported data; the sample not being representative of the quality of life of Venezuelans throughout the country; and the exclusion of Venezuelan migrants without internet access and/or basic computer skills.

Despite the common language, the cultural, historical, and social differences in Spanish-speaking countries can influence an individual perception of quality of life ^23^. We highlight that the Spanish-language version of the WHOQOL-BREF did not undergo cross-cultural adaptation to the specific Venezuelan context. Thus, its application in this population may not have fully captured subjective aspects of self-assessed quality of life. Additional studies, such as those employing qualitative techniques, may capture perceptions beyond what a closed questionnaire can reflect.

## Contributions to the literature

This study explores the gap in Venezuelans’ self-perception of quality of life in Brazil and the contexts that substantially reduce such quality. The findings can support the development of specific and intersectoral strategies directed to Venezuelans in Brazil.
